# The relationship between parenting stress and preschool children’s screen dependence: the chain mediating role of parent–child interaction quality and executive function

**DOI:** 10.3389/fpsyg.2026.1934713

**Published:** 2026-09-04

**Authors:** Huijuan Di, Yuan Liu, Huiqing Li, Hongwei Yao

**Affiliations:** College of Teacher Education, Hebei Normal University, Shijiazhuang, China

**Keywords:** children, executive function, parent–child interaction quality, parenting stress, screen dependence

## Abstract

This study examined the association between parenting stress and preschool children’s screen dependence, with particular attention to the potential serial indirect associations through parent–child interaction quality and children’s executive function. A cross-sectional survey was conducted among 690 parents of children aged 3–6 years. Parenting stress, parent–child interaction quality, children’s executive function, and preschool children’s screen dependence were assessed using validated instruments. Serial mediation analysis was conducted using PROCESS Model 6 with 5,000 bootstrap samples, controlling for children’s gender and age, parental educational level, and annual family income. The results showed that parenting stress was positively associated with preschool children’s screen dependence and negatively associated with parent–child interaction quality and children’s executive function, while parent–child interaction quality was positively associated with executive function. Furthermore, the total indirect effect of parenting stress on screen dependence was significant, including the indirect pathways through parent–child interaction quality, through executive function, and through the serial pathway involving both parent–child interaction quality and executive function. Taken together, these findings indicate that higher parenting stress was associated with greater preschool children’s screen dependence both directly and indirectly through lower parent–child interaction quality and weaker executive function.

## 1 Introduction

In today’s digital age, electronic devices have become an unavoidable part of young children’s daily lives. According to a report by the Organization for Economic Co-operation and Development (OECD, 2023), about 70% of children own their own smartphones, and this rate even exceeds 90% in some countries. In China, nearly 98% of preschool children have access to the internet (Zhang et al., 2018). The widespread and increasingly early use of screens has made screen dependence among young children a growing concern. Screen dependence refers to a condition where children develop an excessive psychological craving for screens and lose control over their screen habits (Bai et al., 2025). Research shows that excessive and early screen use can negatively affect children’s physical development and mental health in various ways (Carson and Janssen, 2012). Physically, it is linked to issues such as obesity, sleep disorders, and vision problems (Mendoza et al., 2007; Magee et al., 2014). Psychologically, it increases the risk of emotional problems like depression and anxiety (Tang et al., 2021; Li et al., 2025; Twenge et al., 2018). Since the home is the primary place where young children use electronic devices, parents play a crucial role in shaping their screen habits (Määttä et al., 2017). Therefore, exploring the risk factors and mechanisms of screen dependence within the family context can provide theoretical support and practical guidance for early prevention and targeted interventions.

### 1.1 Parenting stress and preschool children’s screen dependence

Parenting stress refers to the stress parents experience in the process of carrying out their parenting roles and interacting with their children. It may stem from parents’ own characteristics, difficulties in parent–child interaction, and children’s behaviors (Abidin, 1995). When parents feel that their abilities and available resources are not sufficient to meet parenting demands, parenting stress is likely to occur (Cooper et al., 2009), Recent reports and cross-national evidence further highlight substantial mental health burdens among parents, including parenting stress and parental burnout (Office of the Surgeon General, 2024; Roskam et al., 2021). According to the Family Stress Model, high levels of parenting stress may affect children through parents’ negative emotions and parenting behaviors, thereby increasing the risk of behavioral problems in children (Masarik and Conger, 2017). Specifically, parents under high stress often feel physically and emotionally exhausted. As a result, they may be more likely to use electronic devices as “electronic babysitters” to gain temporary relief, which reduces effective supervision and guidance over children’s screen use. This kind of parental withdrawal may directly create more opportunities for prolonged screen exposure, thus increasing the risk of screen dependence (Parks et al., 2016; Nikken, 2019). Based on this, the present study proposes Hypothesis 1: Parenting stress is positively associated with preschool children’s screen dependence.

### 1.2 The mediating role of parent–child interaction quality

The impact of parenting stress on young children’s screen dependence may not be a simple direct relationship. Instead, it is likely mediated through multiple pathways involving family interaction processes and children’s own cognitive functions. First, parent–child interaction quality may serve as an important mediating mechanism. Parent–child interaction refers to the overall process in which parents and children communicate and influence each other, either directly or indirectly (Tu et al., 2024; Zimmer-Gembeck et al., 2017). Based on parental sensitivity theory, the core feature of this interaction lies in parents’ sensitivity and responsiveness to their children’s needs (De Wolff and Van Ijzendoorn, 1997). This interaction quality is also considered a key foundation for children’s social and emotional development. However, parenting stress may weaken parents’ emotional and cognitive resources. More specifically, parents under high stress are more likely to experience negative emotions such as irritability and anxiety. In interactions with their children, they may show less patience, delayed responses, or even avoidance (Miller-Perrin et al., 2009). Some parents may also rely on electronic devices as a way to escape parenting stress, which may lead them to neglect children’s emotional needs and their need for companionship during development (McDaniel and Radesky, 2020; Anderson and Hanson, 2016; Roskam et al., 2017). In such a low-quality and low-responsiveness interaction environment, children’s needs for emotional attachment may not be adequately met. As a result, screen devices may become an alternative source of emotional comfort and companionship for young children (Kirkorian et al., 2009; Bernier et al., 2016), which may further increase the risk of screen dependence (Ding et al., 2022). Therefore, the present study proposes Hypothesis 2: Parenting stress is indirectly associated with preschool children’s screen dependence through parent–child interaction quality.

### 1.3 The mediating role of executive function

Executive function may also serve as an important mediator in the relationship between parenting stress and preschool children’s screen dependence. Executive function refers to higher-order cognitive processes that regulate and control thoughts and behaviors in order to support goal-directed actions. It mainly includes three components: inhibitory control, cognitive flexibility, and working memory (Friedman and Miyake, 2017). According to self-regulation theory, executive function is the core cognitive basis of self-regulation, and its early development is deeply influenced by the parenting environment (Zimmerman, 2000). Research has shown that parents with high parenting stress are more likely to adopt negative parenting practices, which may hinder children’s autonomous development and, in turn, impair the development of executive function (Blair et al., 2014; Valcan et al., 2018). At the same time, highly stressed parents tend to show lower sensitivity and responsiveness to their children’s emotional needs (Roskam et al., 2017). As a result, children may have fewer opportunities to develop self-regulation through interactions with their parents. Such a low-responsiveness interaction environment is unlikely to provide the emotional support and cognitive scaffolding needed for the development of self-regulation (Bernier et al., 2010; Crandall et al., 2015). Furthermore, a large body of research suggests that weak executive function, especially poor inhibitory control and working memory, is significantly associated with longer screen time and a higher risk of screen dependence in preschool children (Lillard and Peterson, 2011; Hu et al., 2023). More specifically, children with poor inhibitory control may find it difficult to resist the attraction and stimulation of electronic screens (McNeill et al., 2019). Children with weak working memory may also have difficulty maintaining attention in real-world activities and are therefore more easily drawn to screens, which are often characterized by fragmented and short-lasting content (Kostyrka-Allchorne et al., 2017; Lillard and Peterson, 2011). Therefore, the present study proposes Hypothesis 3: Parenting stress is indirectly associated with preschool children’s screen dependence through executive function.

### 1.4 The chain mediating role of parent–child interaction quality and executive function

Notably, parent–child interaction quality and executive function do not exist in isolation. Rather, they may form a sequential mediating chain. Developmental systems theory emphasizes that children’s cognitive abilities are constructed and developed through daily interactions with their primary caregivers. High-quality parent–child interaction provides key experiences such as a secure base, language stimulation, and joint attention, which directly promote the development of the prefrontal cortex and related neural networks in young children, thereby laying a solid foundation for the development of executive function (Bernier et al., 2010; Bernier et al., 2016). In contrast, low-quality parent–child interaction may deprive children of these developmental opportunities. This suggests that higher parenting stress may be associated with poorer parent–child interaction quality, which may in turn be associated with greater executive function deficits. Ultimately, this deficit in self-regulation makes preschool children more vulnerable to screen dependence. Therefore, this study proposes Hypothesis 4: Parenting stress shows a serial indirect association with preschool children’s screen dependence through parent–child interaction quality and executive function.

Although emerging research has begun to examine family pathways linking parenting-related factors with young children’s problematic screen use, important gaps remain. Recent studies have primarily focused on parent-level behavioral and relational processes, such as parental phubbing and parent–child conflict, or have examined parent–child relationships and children’s self-regulatory characteristics in separate roles. For example, Huang et al. (2026) identified parental phubbing and parent–child conflict as sequential pathways linking parenting stress with young children’s problematic media use, whereas Liu et al. (2026) examined the parent–child relationship and effortful control in relation to preschoolers’ screen dependency. However, less is known about whether a family relational process and a child-level cognitive self-regulatory process operate sequentially in the association between parenting stress and preschool children’s screen dependence. The present study addresses this gap by integrating parent–child interaction quality and executive function within the same serial indirect-effects model. This approach links a family-level relational process with a child-level cognitive process and may provide a more integrated account of how parenting stress is statistically associated with preschool children’s screen dependence.

**Figure 1:**
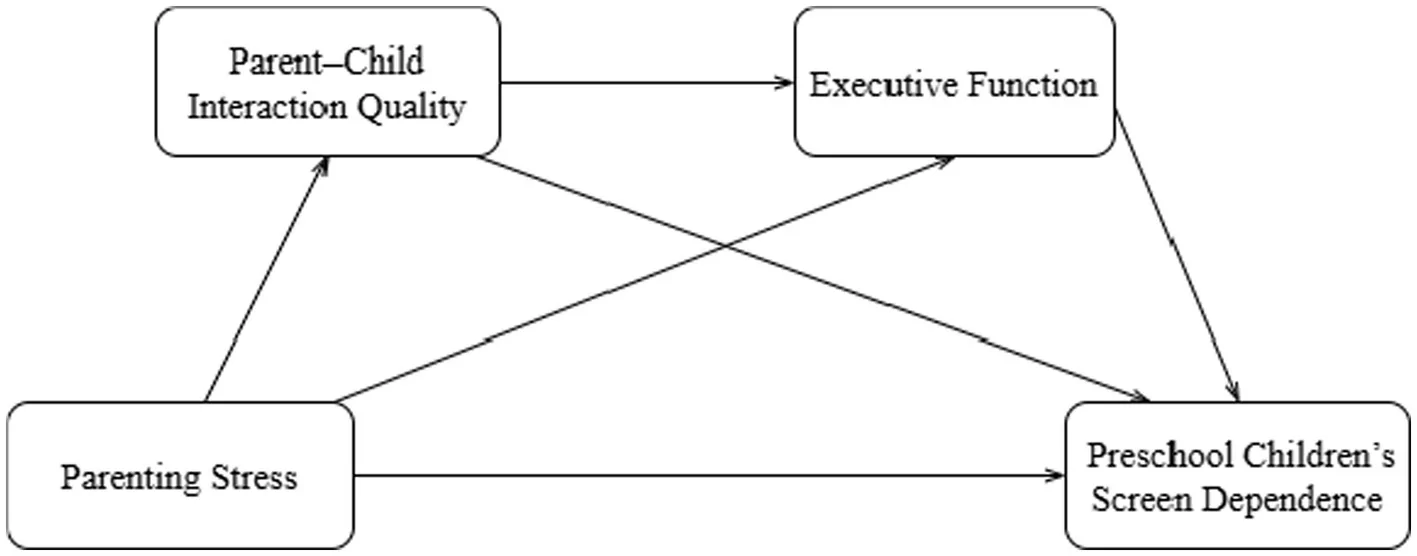
The hypothesized chain mediation model between parenting stress and preschool children’s screen dependence.

In summary, the present study examines the association between parenting stress and preschool children’s screen dependence, with particular attention to the potential serial indirect associations through parent–child interaction quality and executive function. The hypothesized model is shown in Figure 1.

## 2 Methods

### 2.1 Participants

Participants were children aged 3 to 6 years and their parents recruited from four kindergartens in Hebei Province using random sampling. Data were collected via online questionnaires, which were completed by the parents on a voluntary basis. A total of 695 questionnaires were returned. After excluding invalid responses, 690 valid questionnaires remained, yielding an effective response rate of 99.3%. In the present sample, the age distribution was relatively balanced across groups, with children aged 5–6 years accounting for the highest proportion, with boys accounting for 52.75% and girls for 47.25%; parental education was predominantly at the associate or bachelor’s degree level (55.94% in total), and annual household income was concentrated in the range of 60,000–100,000 RMB (32.32%). Detailed demographic characteristics of the sample are presented in Table 1.

**Table 1:** Demographic characteristics of the participants (*N* = 690).

Variables	Category	*n*	%
Children’s age group	3–4 years	206	29.86%
	4–5 years	210	30.44%
	5–6 years	274	39.71%
Children’s gender	Male	364	52.75%
	Female	326	47.25%
Respondent relationship	Father	91	13.19%
	Mother	599	86.81%
Parents’ educational level	Junior high school or below	108	15.65%
	High school or vocational school	181	26.23%
	Associate degree	196	28.41%
	Bachelor’s degree	190	27.54%
	Postgraduate degree or above	15	2.17%
Annual family income (RMB)	<50,000	160	23.19%
	60,000–100,000	223	32.32%
	110,000–150,000	143	20.73%
	160,000–200,000	90	13.04%
	210,000–250,000	26	3.77%
	≥260,000	48	6.96%

### 2.2 Measures

#### 2.2.1 Demographic information

A self-developed demographic questionnaire was used to collect sociodemographic data, including the respondent’s relationship to the child, the children’s age and gender, parents’ educational levels, and annual family income. All information was provided by the parents on a voluntary basis.

#### 2.2.2 Parenting stress scale

This study used the Chinese version of the Parenting Stress Scale (PSS). The original scale was developed by Berry and Jones (1995) and was later translated and adapted for Chinese samples by Cheung (2000). The scale consists of 18 items, 8 of which are reverse scored (Items 1, 2, 5, 6, 7, 8, 17, and 18). Responses are rated on a 5-point Likert scale ranging from 1 (strongly disagree) to 5 (strongly agree). Higher scores indicate greater parenting stress. In this study, the Cronbach’s *α* coefficient for the scale was 0.95.

#### 2.2.3 Preschool children’s screen dependence

This study used the Preschool Screen Dependence Scale developed by Bai et al. (2025). The scale consists of 9 items across three dimensions: emotional symptoms, behavioral symptoms, and pathological symptoms, with 3 items in each dimension. Responses are rated on a 5-point Likert scale ranging from 1 (strongly disagree) to 5 (strongly agree). Higher scores indicate a greater degree of preschool children’s screen dependence. In this study, the Cronbach’s *α* coefficient for the scale was 0.91.

#### 2.2.4 Parent–child interaction quality

This study used the Brigance Parent–Child Interactions Scale (BPCIS), developed by Glascoe and Leew (2010). The scale includes 18 items across three dimensions: closeness, interaction, and responsiveness, with 6 items in each dimension. Items are rated on a 5-point Likert scale, ranging from 1 (never) to 5 (always). Higher scores indicate better parent–child interaction quality. In the present study, the Cronbach’s *α* for this scale was 0.94.

#### 2.2.5 Executive function

Executive function was measured using the Childhood Executive Functioning Inventory (CHEXI), which is based on Barkley’s hybrid model and was originally developed by Thorell and Nyberg (2008). The scale has been translated into Chinese (Thorell et al., 2013) and has demonstrated good reliability and validity among Chinese preschool children (Wei et al., 2018). The inventory consists of 24 items covering four dimensions: working memory, planning, inhibitory control, and regulation, with six items in each dimension. Items are rated on a 5-point Likert scale. In the original scoring system, higher scores indicate greater executive function difficulties and thus weaker executive functioning. For ease of interpretation in the present study, all items were reverse-scored prior to calculating the dimension and total scores, such that higher scores indicated better executive functioning. In the present sample, Cronbach’s *α* for the scale was 0.97.

### 2.3 Data analysis

Data were analyzed using IBM SPSS Statistics 27.0 and AMOS 29.0. Confirmatory factor analysis was conducted in AMOS to assess the measurement structure and potential common method bias. Serial mediation analyses were performed using Model 6 of the PROCESS macro for SPSS (Hayes, 2013), with 5,000 bootstrap samples used to estimate 95% confidence intervals for the indirect effects. Children’s gender and age, parental educational level, and annual family income were included as covariates because these sociodemographic characteristics may be associated with parenting stress and children’s media-use environments.

## 3 Results

### 3.1 Common method bias assessment

Harman’s single-factor test was used to test for common method bias (Zhou and Long, 2004). The results showed that 10 factors with eigenvalues greater than 1 were extracted, and the first unrotated factor explained only 27.73% of the total variance. Since this is below the 50% critical threshold, the preliminary result did not indicate a dominant single common factor.

Given the methodological limitations of Harman’s single-factor test, confirmatory factor analysis (CFA) was further conducted. As shown in Table 2, the hypothesized four-factor model demonstrated an acceptable fit to the data, *χ*^2^(48) = 231.160, *χ*^2^/df = 4.816, GFI = 0.945, AGFI = 0.911, CFI = 0.969, TLI = 0.957 and RMSEA = 0.074. In contrast, the single-factor model, in which all observed indicators were loaded onto one latent factor, demonstrated substantially poorer fit, *χ*^2^(54) = 2704.620, *χ*^2^/df = 50.086, GFI = 0.562, AGFI = 0.367, CFI = 0.547, TLI = 0.446, and RMSEA = 0.267. The difference between the two models was statistically significant, Δ*χ*^2^(6) = 2,473.460, *p* < 0.001. These findings indicate that the covariation among the study variables cannot be adequately explained by a single common factor, suggesting that a dominant common method factor was unlikely to account for the observed associations.

**Table 2:** Comparison of confirmatory factor analysis models.

Model	*χ* ^2^	df	*χ*^2^/df	CFI	TLI	GFI	AGFI	RMSEA
Single-factor model	2,704.620	54	50.086	0.547	0.446	0.562	0.367	0.267
Four-factor model	231.160	48	4.816	0.969	0.957	0.945	0.911	0.074

### 3.2 Descriptive statistics and correlations

The descriptive statistics (means and standard deviations) and Pearson correlations for all variables are presented in Table 3. The results of the Pearson correlation analysis revealed that parenting stress, parent–child interaction quality, children’s executive function, and preschool children’s screen dependence were significantly correlated with each other. Specifically, parenting stress was significantly positively correlated with preschool children’s screen dependence (*r* = 0.438, *p* < 0.001), and significantly negatively correlated with parent–child interaction quality (*r* = −0.439, *p* < 0.001) and children’s executive function (*r* = −0.401, *p* < 0.001). Preschool children’s screen dependence was significantly negatively correlated with parent–child interaction quality (*r* = −0.360, *p* < 0.001) and children’s executive function (*r* = −0.454, *p* < 0.001). Parent–child interaction quality was significantly positively correlated with children’s executive function (*r* = 0.355, *p* < 0.001).

**Table 3:** Descriptive statistics and correlations among variables (*N* = 690).

Variables	*M*	SD	1	2	3	4	5	6	7	8
1. Child gender	–	–	–							
2. Child age	–	–	−0.057	–						
3. Parental education	–	–	−0.012	−0.001	–					
4. Family income	–	–	−0.024	−0.032	0.129***	–				
5. Parenting stress	37.581	11.370	−0.039	0.072	−0.093*	−0.191***	–			
6. Preschool children’s screen dependence	24.380	8.246	−0.054	0.094*	−0.081*	−0.057	0.438***	–		
7. Parent–child interaction quality	69.793	9.264	0.057	−0.123**	0.143***	0.118**	−0.439***	−0.360***	–	
8. Executive function	80.494	15.360	0.069	0.020	0.037	0.042	−0.401***	−0.454***	0.355***	–

*p < 0.05, **p < 0.01, ***p < 0.001.

### 3.3 Chain-mediating role

The mediation analysis was conducted using Model 6 in the PROCESS macro for SPSS 27.0 developed by Hayes (2013). Children’s gender and age, parental educational level, and annual family income were included as covariates. Parenting stress was entered as the independent variable, preschool children’s screen dependence as the dependent variable, and parent–child interaction quality and children’s executive function as the two sequential mediators.

As shown in Table 4, after controlling for children’s gender and age, parental educational level, and annual family income, parenting stress was significantly and positively associated with preschool children’s screen dependence (*β* = 0.434, *p* < 0.001) and significantly and negatively associated with parent–child interaction quality (*β* = −0.417, *p* < 0.001). Parenting stress was also significantly and negatively associated with children’s executive function (*β* = −0.312, *p* < 0.001), whereas parent–child interaction quality was significantly and positively associated with children’s executive function (*β* = 0.232, *p* < 0.001). In the final model, parenting stress remained significantly and positively associated with preschool children’s screen dependence (*β* = 0.255, *p* < 0.001). Parent–child interaction quality was significantly and negatively associated with screen dependence (*β* = −0.129, *p* < 0.001), and children’s executive function was also significantly and negatively associated with screen dependence (*β* = −0.307, *p* < 0.001). Parental educational level was positively associated with parent–child interaction quality (*β* = 0.101, *p* = 0.003), whereas annual family income was not significantly associated with any of the modeled outcomes.

**Table 4:** Regression analyses for the serial mediation model (*N* = 690).

Outcome variable	Predictor	*B*	SE	*β*	*t*	*R* ^2^	Adjust. *R*^2^	*F*
Screen dependence	Child gender	−0.543	0.566	−0.033	−0.960	0.200	0.194	34.143***
	Child age	0.617	0.342	0.062	1.805			
	Parental education	−0.340	0.262	−0.045	−1.300			
	Family income	0.192	0.204	0.033	0.945			
	Parenting stress	0.315	0.025	0.434	12.398***			
Parent–child interaction quality	Child gender	0.682	0.631	0.037	1.082	0.214	0.208	37.155***
	Child age	−1.005	0.381	−0.090	−2.640**			
	Parental education	0.858	0.292	0.101	2.943**			
	Family income	0.153	0.227	0.023	0.673			
	Parenting stress	−0.340	0.028	−0.417	−12.014***			
Executive function	Child gender	1.432	1.050	0.047	1.364	0.210	0.203	30.238***
	Child age	1.352	0.636	0.073	2.125*			
	Parental education	−0.276	0.488	−0.020	−0.567			
	Family income	−0.416	0.377	−0.038	−1.102			
	Parenting stress	−0.422	0.052	−0.312	−8.142***			
	Parent–child interaction quality	0.384	0.064	0.232	6.039***			
Screen dependence	Child gender	−0.186	0.529	−0.011	−0.351	0.306	0.298	42.870***
	Child age	0.660	0.322	0.066	2.053*			
	Parental education	−0.233	0.246	−0.031	−0.947			
	Family income	0.151	0.190	0.026	0.795			
	Parenting stress	0.185	0.027	0.255	6.771***			
	Parent–child interaction quality	−0.115	0.033	−0.129	−3.504***			
	Executive function	−0.165	0.019	−0.307	−8.539***			

*B*, unstandardized coefficient; SE, standard error; *β*, standardized coefficient. *p < 0.05, **p < 0.01, ***p < 0.001.

A bootstrap analysis was further conducted to examine the statistical significance of the indirect associations, and the results are presented in Table 5. After controlling for children’s gender and age, parental educational level, and annual family income, the total effect of parenting stress on preschool children’s screen dependence was 0.315, with a 95% confidence interval of [0.265, 0.365]. After parent–child interaction quality and children’s executive function were included in the model, the direct effect remained significant, with an effect size of 0.185 and a 95% confidence interval of [0.131, 0.239], accounting for 58.72% of the total effect. The total indirect effect was 0.130, with a 95% bootstrap confidence interval of [0.092, 0.171], accounting for 41.28% of the total effect.

**Table 5:** Total, direct, and indirect effects of parenting stress on screen dependence (*N* = 690).

Effect pathway	Effect	SE	BootLLCI	BootULCI	Percentage
Total effect	0.315	0.025	0.265	0.365	100.00%
Direct effect	0.185	0.027	0.131	0.239	58.72%
Total indirect effect	0.130	0.020	0.092	0.171	41.28%
PS → PCIQ → SD	0.039	0.012	0.016	0.064	12.43%
PS → EF → SD	0.069	0.015	0.042	0.102	22.03%
PS → PCIQ → EF → SD	0.021	0.005	0.012	0.032	6.82%

PS, parenting stress; PCIQ, parent–child interaction quality; EF, children’s executive function; SD, preschool children’s screen dependence.

**Figure 2:**
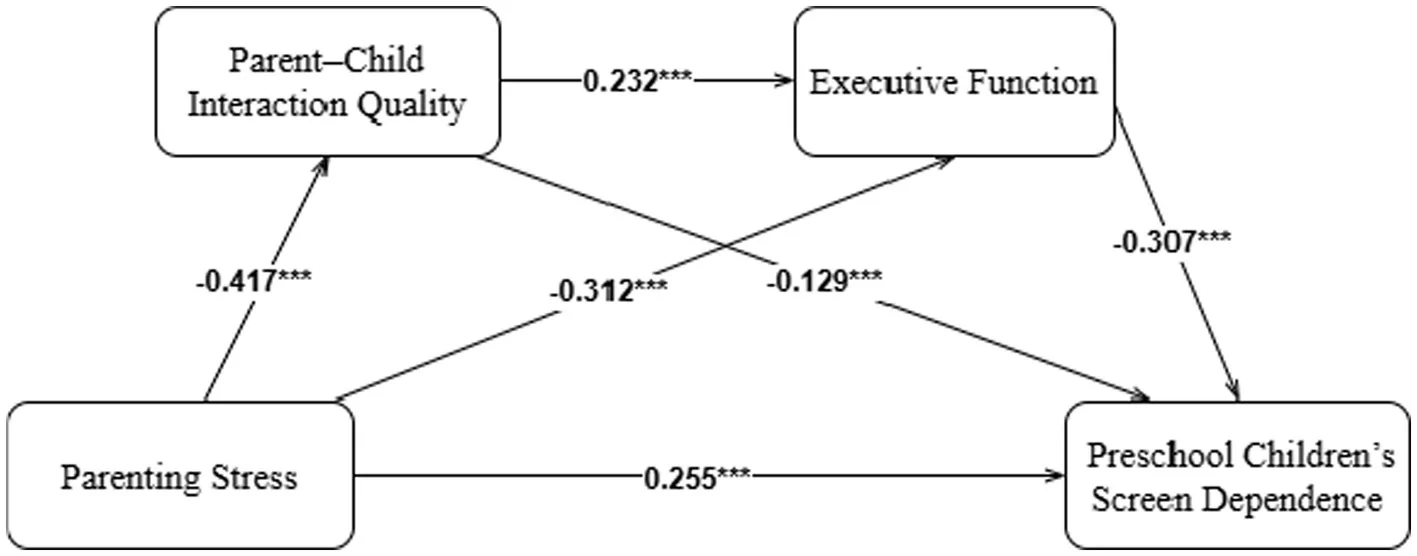
Standardized path coefficients for the chain mediation model. ***p < 0.001.

The total indirect effect consisted of three specific pathways. First, the indirect association through parent–child interaction quality (PS → PCIQ → SD) was significant, with an effect size of 0.039 and a 95% bootstrap confidence interval of [0.016, 0.064], accounting for 12.43% of the total effect. Second, the indirect association through children’s executive function (PS → EF → SD) was significant, with an effect size of 0.069 and a 95% bootstrap confidence interval of [0.042, 0.102], accounting for 22.03% of the total effect. Third, the serial indirect association through parent–child interaction quality and children’s executive function (PS → PCIQ → EF → SD) was also significant, with an effect size of 0.021 and a 95% bootstrap confidence interval of [0.012, 0.032], accounting for 6.82% of the total effect. None of the 95% bootstrap confidence intervals included zero, indicating that all three specific indirect associations were statistically significant. The final model is shown in Figure 2.

## 4 Discussion

### 4.1 The relationship between parenting stress and preschool children’s screen dependence

The present study found that parenting stress was significantly and positively associated with preschool children’s screen dependence. Specifically, higher parenting stress is associated with a greater risk of screen dependence in children. This finding is consistent with previous studies (McDaniel and Radesky, 2020). According to the Family Stress Model, parents experiencing high levels of stress tend to exhibit negative parenting behaviors, which in turn may be associated with a greater likelihood of problem behaviors in children (Neece et al., 2012). Due to constraints on their time and energy, parents under high stress often struggle to provide high-quality parent–child interaction and companionship. Consequently, this may result in less effective monitoring and guidance regarding their children’s screen use. In such contexts, when children’s emotional needs remain unmet through parent–child interaction, they often turn to electronic devices for compensatory comfort, which may contribute to the reinforcement of screen-dependent behaviors (Parks et al., 2016; Nikken, 2019).

### 4.2 The mediating roles of parent–child interaction quality

The results indicated a significant indirect association between parenting stress and preschool children’s screen dependence through parent–child interaction quality. This finding is consistent with previous research (Yu et al., 2025). Specifically, parenting stress was significantly and negatively associated with parent–child interaction quality, and higher quality of parent–child interaction was associated with lower levels of screen dependence in children. Parents experiencing lower levels of parenting stress typically possess sufficient time and energy to actively engage in high-quality interactions with their children, which take various forms such as shared reading, joint play, and emotional communication (Miller-Perrin et al., 2009; Nan and Tian, 2025; Allen and Marshall, 2011). Such interactions not only occupy children’s leisure time—thereby reducing their opportunities to access electronic devices—but also fulfill their emotional needs, which decreases their tendency to seek compensatory comfort from screens. Thus, higher-quality parent–child interaction may represent a potentially protective relational context associated with lower levels of children’s screen dependence.

### 4.3 The mediating roles of executive function

The present study identified a significant indirect association between parenting stress and preschool children’s screen dependence through children’s executive function, which is consistent with previous research (Blair et al., 2014; Valcan et al., 2018; Bernier et al., 2010). Hanlin and Zhou (2024) noted that parenting behaviors play an important role in the development of children’s executive function. Because parenting stress is closely related to parenting practices, it may also be associated with children’s executive functioning. In families experiencing high levels of parenting stress, parents may be more likely to adopt negative parenting practices and may have greater difficulty providing positive responses and emotional support to their children, which may in turn hinder the development of executive function (Zhang et al., 2020). Children with weaker executive function, particularly poorer inhibitory control, may be more easily attracted to electronic devices and may have greater difficulty regulating their own screen use, thereby increasing the likelihood of screen dependence (Kostyrka-Allchorne et al., 2017; Lillard and Peterson, 2011). Therefore, lower levels of executive function may represent an important indirect pathway linking parenting stress with greater screen dependence among preschool children.

### 4.4 The chain mediating roles of parent–child interaction quality and executive function

The present study identified a significant serial indirect association linking parenting stress with preschool children’s screen dependence through parent–child interaction quality and children’s executive function. Stressed parents often struggle to allocate sufficient time for their children and may exhibit a lack of sensitivity and responsiveness during their limited interactions. Consequently, they tend to neglect their children’s emotional needs and desire for autonomous exploration, failing to provide stable autonomy support. Such low-quality interactions may provide fewer opportunities for children to practice rule compliance, delay of gratification, and problem-solving, which in turn may be associated with weaker executive functioning (Valcan et al., 2018; Bernier et al., 2010; Crandall et al., 2015). In contrast, when parents invest more time and energy into parent–child interactions, they create a secure and supportive developmental environment by responding to children’s needs, encouraging autonomous exploration, and engaging in rule-based games together. Within these intimate interactions, children can gradually acquire self-regulation strategies, which may support the development of executive functioning (Hughes and Devine, 2019; Soydan et al., 2025).

### 4.5 Conclusions, limitations, and implications

This study provides evidence of a significant association between parenting stress and preschool children’s screen dependence and identifies significant indirect associations involving parent–child interaction quality and children’s executive function. By integrating a family relational process with a child-level cognitive self-regulatory process, the findings extend previous research on family correlates of young children’s screen dependence.

From a practical perspective, the findings suggest that efforts to address preschool children’s problematic screen use may benefit from considering the broader family context rather than focusing solely on restricting children’s screen time. For parents experiencing high levels of parenting stress, family-support programs could provide stress-management resources and encourage regular screen-free parent–child activities, such as shared reading, joint play, and responsive conversation. Such activities may provide opportunities for emotional connection and for children to practice attention, inhibitory control, and rule-following in everyday contexts. Preschool teachers, psychologists, and family-service practitioners may also consider parenting stress, parent–child interaction quality, and children’s self-regulatory difficulties when identifying families that may need additional support.

Several limitations should be acknowledged. First, although children’s gender, age, parental education, and annual household income were controlled for as covariates, these variables themselves have theoretically meaningful associations with the core variables. Correlational analyses revealed that parental education and household income were significantly negatively correlated with parenting stress and significantly positively correlated with parent–child interaction quality, consistent with the Family Stress Model, which posits that a lack of socioeconomic resources may exacerbate parenting stress and undermine interaction quality (Masarik and Conger, 2017). In addition, children’s age was significantly negatively correlated with parent–child interaction quality and positively correlated with screen dependence, possibly reflecting that older children face more complex screen-use contexts and greater difficulties in parental monitoring (Määttä et al., 2017). However, the present sample was predominantly composed of parents with associate or bachelor’s degrees and middle-level household income, and this socioeconomic homogeneity may limit the generalizability of our conclusions to populations with lower educational attainment, lower income, or from rural areas. Future research should include more diverse samples and further examine whether these variables play moderating roles in the model, so as to clarify the boundary conditions and population specificity of this mechanism. Second, the majority of respondents were mothers, with an insufficient sample size of fathers. Given that there may be gender differences in parenting stress and parenting behaviors between mothers and fathers, whether the conclusions of this study are equally applicable to fathers remains to be further verified. Future research should recruit a more balanced sample of both parents and examine whether parental gender plays a moderating role. Third, children’s executive function was measured solely through parental ratings, without the use of behavioral assessments such as laboratory tasks. Future research could combine parent reports with objective behavioral measures of inhibitory control, working memory, and cognitive flexibility to reduce same-respondent bias and provide a more comprehensive evaluation of children’s executive functioning.

In conclusion, parenting stress was significantly positively correlated with preschool children’s screen dependence and significantly negatively correlated with children’s executive function; parent–child interaction quality was significantly negatively correlated with parenting stress and preschool children’s screen dependence but significantly positively correlated with children’s executive function; and parent–child interaction quality and children’s executive function played a serial mediating role in the relationship between parenting stress and preschool children’s screen dependence.

## Data Availability

The raw data supporting the conclusions of this article will be made available by the authors, without undue reservation.
